# Building connection: overdose survivors’ and professional service providers’ perspectives on immediate post-overdose care

**DOI:** 10.1016/j.drugpo.2025.104948

**Published:** 2025-08-09

**Authors:** Ranjani K. Paradise, Simeon D. Kimmel, Alykhan Nurani, Jeff Desmarais, Shannon O’Malley, Alexander Y. Walley, Andres Hoyos-Cespedes, Jaylen Clarke, Sunday Taylor, Daniel Dooley, Angela R. Bazzi

**Affiliations:** a Institute for Community Health, Malden, MA, United States; b Tufts University School of Medicine, Boston, MA, United States; c Cambridge Health Alliance, Cambridge, MA, United States; d Boston Medical Center, Boston, MA, United States; e Boston University Chobanian and Avedisian School of Medicine, Boston, MA, United States; f Boston Public Health Commission, Boston, MA, United States; g Massachusetts Department of Public Health, Bureau of Substance Addiction Services, Boston, MA, United States; h Massachusetts Department of Public Health, Office of Population Health, United States; i Herbert Wertheim School of Public Health, University of California San Diego, La Jolla, CA, United States; j Boston University School of Public Health, Boston, MA, United States

**Keywords:** Drug overdose, Naloxone, Opioids, Psychological distress, Harm reduction, Referral and consultation

## Abstract

**Background:**

Nonfatal overdose is a risk factor for future fatal overdose and represents a critical touchpoint for engaging survivors and making connections to treatment and harm reduction resources. The aim of this study was to understand survivors’ experiences and preferences in the immediate post-overdose period, and to elucidate survivors’ and professionals’ perspectives on improving care provision at this interaction point.

**Methods:**

In 2020–2021, we conducted semi-structured qualitative interviews with opioid overdose survivors (*n* = 59) and professionals (*n* = 28) who respond to overdoses or interact with survivors in Boston, MA. When reviewing coded data early in the analytical process, we identified a strong emphasis on the importance of immediate post-overdose experiences in influencing engagement in care. Subsequent in-depth analysis then identified common experiences and factors related to service engagement in this acute time period.

**Results:**

Among 59 overdose survivors, most identified as Black or Latinx (70 %) due to purposive sampling. Most were also unhoused (75 %) and reported at least three past-year overdoses (69 %). Many participants described intense physical pain and/or emotional distress immediately following overdose reversal, which reduced their desire and ability to engage with service providers. Several experienced disrespect and stigma from overdose responders, which negatively impacted their experience. However, some participants expressed wanting to be offered services immediately post-overdose, stating that providers should always *“extend the branch”*. Professionals reinforced survivors’ perspectives, explaining how trauma and stigma reduce survivors’ willingness to accept service information and referrals; they also highlighted systemic challenges in standard overdose response processes that impede effective engagement.

**Conclusion:**

To better engage survivors, overdose response processes should prioritize survivors’ physical and emotional comfort and seek to build trust by utilizing person-centered, trauma-informed, and non-stigmatizing approaches.

## Introduction

Overdose killed more than 107,000 people in the United States in 2023. Non-fatal overdose is a risk factor for future fatal overdose and often results in activation of emergency medical services (EMS), representing an important opportunity for connecting survivors to treatment and other services (Caudarella et al., 2016; Darke et al., 2011; Krawczyk et al., 2020; Larochelle et al., 2019). However, the majority of survivors are not successfully connected to evidence-based substance use disorder (SUD) treatment or harm reduction services following overdose reversal (Alinsky et al., 2020; Crystal et al., 2022; Dooley, 2019; Frazier et al., 2017; Larochelle et al., 2018; Pollini et al., 2006; Watson et al., 2024). To address this gap, post-overdose interventions to increase evidence-based SUD treatment and harm reduction service provision have expanded. For opioid use disorder, treatment with medications such as methadone and buprenorphine is considered the gold standard, with strong evidence of effectiveness for reducing mortality and reducing other opioid use, among other positive outcomes (National Academies of Sciences et al., 2019). In emergency departments (EDs), there have been broad efforts to provide overdose survivors with medications for opioid use disorder (MOUD) and link them to follow-up care, and some EDs offer harm reduction supplies (e.g., naloxone, sterile injection equipment) and peer support services (Bagley et al., 2019; Beaudoin et al., 2022; Jacka et al., 2022; Jiao et al., 2024; Samuels et al., 2019; Treitler et al., 2024; Watson et al., 2024). Some EMS first responders also provide buprenorphine to overdose survivors in order to initiate evidence-based treatment without requiring the patient to accept transport to a medical facility, which can be a barrier (Belden et al., 2024; Carroll et al., 2023; Hern et al., 2023). In the days following these acute interactions, public health and public safety organizations are increasingly collaborating to conduct post-overdose outreach and offer survivors and their families overdose education, naloxone, and referrals to treatment services (Bagley et al., 2019; Bailey et al., 2023; Carroll et al., 2023; Champagne-Langabeer et al., 2020; Kimmel et al., 2023; Langabeer et al., 2020, 2021; Xuan et al., 2023).

Although these interventions hold promise, site-level variation in outcomes and gaps in survivors’ service engagement persist (Beaudoin et al., 2022; Langabeer et al., 2020; Treitler et al., 2024; Watson et al., 2024; Winhusen et al., 2020). Further, while naloxone is lifesaving, the experience of overdose reversal can be unpleasant for many survivors due to precipitated withdrawal and proximity to rescuers’ anxiety and fear, which can generate anger and tension for both parties (Bowles et al., 2020; Brandt et al., 2022; Macmadu et al., 2022; Neale et al., 2020; Parkin et al., 2020).

Improving interventions to better engage survivors in treatment and harm reduction services requires an enhanced understanding of survivor and service provider experiences on engagement challenges and opportunities in the time period immediately following overdose, before individuals are lost to follow-up care. As this acute post-overdose time period emerged as highly significant in qualitative interviews we conducted with overdose survivors and professional key informants in Boston, Massachusetts, we conducted an in-depth analysis to illuminate survivors’ experiences and survivors’ and professionals’ perspectives on how to improve care provision and linkages at this critical point.

## Methods

### Study design and sample

This analysis utilizes data from the Boston Overdose Linkage to Treatment Study (BOLTS), which used surveys and semi-structured qualitative interviews in 2020–2021 to explore racial and ethnic inequities in access to SUD treatment and other services following opioid overdoses in Boston (Paradise et al., 2024). BOLTS included two participant groups: overdose survivors and professionals. Eligible overdose survivors were ≥18 years old, resided in Boston, experienced an opioid overdose within the past three months, spoke English or Spanish, and identified as Hispanic or Latino/a/x, Black, and/or White. As described previously (Paradise et al., 2024, 2023), we partnered with community-based organizations to recruit participants, and used purposive sampling to ensure a racially and ethnically diverse sample. Eligible professionals were ≥18 years old and had professional or community leadership experience that enabled them to speak about barriers and facilitators to SUD treatment access, equity, and engagement among overdose survivors. This included experience personally responding to overdoses, working in relevant policy or advocacy roles, and/or providing medical, SUD, or social services to overdose survivors in Boston. We refer to this group as “professionals” throughout the manuscript for brevity. We identified an initial list of potential professionals and then used purposive and snowball sampling techniques to recruit individuals representing a range of roles and settings. All participants provided verbal informed consent. Overdose survivors received $50 and professionals received $25 (gift cards) as compensation for their time. The Boston University Medical Center Institutional Review Board approved all study protocols.

### Data collection

Data collection occurred between September 2020-September 2021. For overdose survivors, trained interviewers conducted study visits by videoconference or in person, starting by administering a brief survey on socio-demographics and drug use and overdose histories. Interviewers then conducted qualitative interviews using a semi-structured interview guide (Paradise et al., 2024, 2023). For professionals, study visits followed similar procedures and were conducted via videoconference. Interview guides contained open-ended questions and probes designed to explore experiences and perspectives related to overdose, SUD treatment, and harm reduction services in Boston, focusing on formalized care systems and professional providers. For overdose survivors, examples of open-ended questions relevant to this analysis included, *“Can you tell me about that overdose and what happened that day?”; “How did you recover from that overdose?”*; and *“What was it like for you when you were getting treated for the overdose?”* For professionals, examples of relevant questions included, *“In your experience, what types of services do people most commonly engage in after an overdose?”; “How do people get connected with these services?*”; *“What factors influence people’s decisions about whether or not to seek treatment for opioid misuse after an overdose?”*; and *“What influences people’s decisions about whether or not to seek harm reduction services after an overdose?”* Interviews were audio-recorded and professionally transcribed, and transcripts were reviewed to ensure accuracy and de-identification.

### Data analysis

We used an iterative, collaborative codebook development process (DeCuir-Gunby et al., 2011; MacQueen et al., 1998) engaging multiple individuals across our interdisciplinary team in developing deductive and inductive codes, resulting in separate codebooks for each participant group (i.e., survivors and professionals). First, team members who were involved in study design and data collection developed preliminary codes, which they applied to transcripts independently. They then met to discuss code application and refine codes, repeating this process until reaching consensus on final codebooks for each participant group. We then applied final codes to transcripts using Dedoose, meeting weekly to review coding program and consistency and resolve discrepancies through a group consensus. Next, we used the framework analysis method to summarize coded data at the participant level and develop higher-level analytical memos (Gale et al., 2013), as previously described (Paradise et al., 2024). The study PI and a co-investigator reviewed code application and analytic memos to ensure fidelity and accuracy and identify emergent themes. During this review process, the investigators noticed a strong emphasis that many participants placed on the importance of the immediate post-overdose experience in influencing service engagement. Then, we conducted a more in-depth analysis of codes and framework memos related to this acute time period to identify common experiences and related factors that participants perceived as affecting engagement with service providers. Over a series of meetings, we collaboratively reviewed, triangulated, and interpreted these themes, finally selecting representative quotes to illustrate key findings in the next section.

## Results

### Participant characteristics

Among 59 overdose survivor participants (Table 1), the majority (*n* = 41; 70 %) identified as cisgender male, with roughly equal distribution across racial/ethnic categories (23 identified as Hispanic or Latino/a/x [39 %], 18 as non-Hispanic Black [31 %], and 18 as non-Hispanic White [31 %]). Most had been incarcerated (*n* = 50; 85 %) and were experiencing homelessness (*n* = 44; 75 %). Twenty-six (44 %) survivors reported their physical health as “Excellent” or “Good,” versus 15 (25 %) for mental health. The majority had experienced three or more overdoses in the past year (*n* = 41; 69 %).

Among 28 professionals (Table 2), 12 identified as non-Hispanic White (43 %), eight as non-Hispanic Black (29 %), and seven as Hispanic or Latino/a/x (25 %). The most common organizations represented were outpatient SUD treatment providers (*n* = 7; 25 %), public health officials (*n* = 6; 21 %), and detoxification or residential SUD treatment providers (*n* = 5; 18 %). Common roles were service providers in managerial (*n* = 10; 36 %) or front line positions (*n* = 6; 21 %), physicians (*n* = 4; 14 %), first responders including EMS providers (*n* = 3, 11 %), and advocates/activists (*n* = 3, 11 %).

### Key findings

Qualitative analysis identified themes related to survivors’ experiences immediately following overdose and factors that affect engagement with service providers at this critical touchpoint. Because overdose survivors and professionals represent different perspectives, we present the findings from each participant group separately for clarity, beginning with overdose survivors (Section 3.2.1 and 3.2.2), followed by professionals (Section 3.2.3).

#### Survivors’ physical and emotional reactions immediately after overdose

Overdose survivors recounted how they felt immediately after their most recent overdose, with many describing intense physical distress, including pain, agitation, and *“awful”* withdrawal symptoms related to naloxone (Narcan) treatment. For some, physical side effects of naloxone made them feel *“like death.”* A Black male in his thirties recalled, *“I was gagging and throwing up left [and] right. Sweating profusely, runny nose…It wasn’t good…pain in my stomach…”* Another participant (Latino male, thirties) explained, *“You always feel like shit after you get Narcan[ed]; you feel sick…agitated, sweating.”* Due to these physical side effects, a Black male in his forties summarized that naloxone *“just takes a lot out of you.”*

Several participants also experienced strong emotional reactions post-overdose, including feelings of sadness, anger, worthlessness, shame, and guilt. For example, a White male in his forties described sadness and self-blame about not maintaining his sobriety: *“I was sad… because I relapsed and I really wanted to stay sober, [but] I put myself there… I was definitely depressed by the whole situation.”* Another participant (Black male, thirties) described his sense of *“letting down”* people in his life:

My first thoughts were like, “fuck,” you know, then, “not again.” I looked down on myself…It’s just a lot of guilt because…I’ve got a lot of people out there that love and care about me, and I know it’s like…playing with [my] life.

Another survivor (Latino male, thirties), who described himself as having *“chronic separation anxiety,”* emphasized the distress he felt when he awoke surrounded by firefighters and police rather than the people he had been with earlier:

I looked around. I was abandoned; the girls I was with [weren’t] there anymore. I felt betrayed, I felt ashamed, I felt stupid, I felt worthless, I felt like an idiot, I felt angry…then I felt embarrassed. I began coming back to life being embarrassed.

Because of these physical and emotional reactions, some participants explained how they were not ready or in the right frame of mind to discuss or engage in treatment or other resources recommended by service providers immediately following overdose. A White male in his thirties explained:

You’re so sick after that, [treatment] is the last thing on your mind, like, just don’t ask me anything, you know? … Maybe the next day, you’d be up for thinking about [treatment] a little more, but usually not right after [the overdose]. Some people maybe, but not me.

Others talked about how feeling upset and sick made them want to immediately leave the scene where they overdosed rather than talking with first responders or service providers, as a Latino male in his thirties described:

You wake up with your pants all broken down with scissors or whatever… then [first responders] are asking you, “Hey, what’s your name? What happened?” [But] you’re not in the mood; you’re sick, you woke up sick, dying…like, all you want is drugs. That’s all. When you wake up, because Narcan takes away every bit of heroin you got [in] your body, you don’t want to hear none of that. So, I wake up, and I start arguing, like, “I want to leave, I want to get out of here!” It’s always like that, always, always, always.

#### Survivors’ perspectives on interpersonal interactions with overdose responders

Overdose survivors elaborated on the nature of their interactions with personnel who responded to their overdoses, including first responders, harm reduction program staff, and hospital staff. Many participants had positive interactions and felt that responders treated them respectfully, such as a Latino male in his fifties, who said, *“The paramedics [and] the ambulance staff are really kind, they’re very attentive…I’m grateful to those people.”* Similarly, a White male in his forties described an interaction with a nurse from a harm reduction program who responded to his overdose, saying, *“I felt like I was being treated very well. The nurse was very understanding [and] definitely cared.”*

However, others felt disrespected or stigmatized when interacting with responders, often referring to hospital or EMS staff. For example, a Black female in her twenties spoke about experiencing stigma and “*lack of respect”* when being treated in a local hospital following a recent overdose, explaining that *“especially [when] they know you’re an addict, they definitely don’t give you the full respect you deserve.”* A Black male in his thirties also reflected on negative experiences with hospital staff, describing lack of sensitivity and empathy:

Honestly, I feel like if they [could] just be a little bit more sensitive to addicts who just literally almost died and who are homeless and who are going through fucking hell? If they just…put themselves in our shoes [and] realized that we’re people, too, it’d be a little bit more pleasant place to visit, you know?

Several participants described professionals who neglected to offer them referrals or information about available services, or did not do so in a caring or supportive manner, reinforcing their feelings of neglect or disregard. For these participants, it was important to be offered help, regardless of their desire to engage in treatment or services at that moment, as explained by a Latino male in his fifties:

Don’t you think you should offer me detox or something? Like, I just OD’d here…if [an overdose] happens, once you’re up and able, [usually the responder says], “There, he’s up, we got to go…” I know you can’t spend two or three hours with a person, but at least give them 10–15 min [of] conversation, like, “Hey, listen, do you need to go to detox?…Do you want help?” Extend the branch…If he don’t want the branch, if he swipes it away, okay, at least you tried to…get [him] down the right road.

Similarly, another participant (Latino male, thirties) described what he wished the hospital staff who treated his overdose had said after he was stabilized. Though this survivor already had significant knowledge about the local landscape of SUD services, he wanted staff to demonstrate support by initiating a conversation about treatment options:

They said I was ready to go, but they could have said, “We see you coming in here…we see you walking out. We see you around the neighborhood. Do you think it’s time to cool your heels a little [and] we can work something out?…We’ll keep you for 24 h. We have an addiction team [to treat you] so you’re not sick, we’ll get you on methadone, and possibly have you talk to our [linkage] team…to discuss treatment options…If you’re not good with inpatient, we [could] set up outpatient…” They could have gone that way [but] they didn’t.

For these participants, if responders *“could have said more”* or offered more time or attention, it could have built their trust regardless of their immediate willingness or ability to engage in treatment services.

#### Professionals’ perspectives on challenges in overdose response

Based on their experiences directly responding to overdoses or interacting with survivors, professionals shared their perspectives on the factors that shape survivors’ desire and ability to engage with SUD treatment or other services immediately following overdose.

#### Experiences of trauma, stigma, and judgement deter service engagement.

Several professionals emphasized that overdose is a traumatic event, echoing survivors’ descriptions (Section 3.2.1) of intense physical and emotional distress immediately following overdose reversal, and explaining that survivors are often focused on managing immediate needs rather than thinking about treatment options. As an SUD treatment clinician explained, *“If you wind up in an emergency department because of an overdose…you’re not necessarily like, ‘I need to get linked to services.’ [Instead] you’re like, ‘I need this episode in my life to be over right now and I need to go back to normal.’”* A harm reduction program manager elaborated on how survivors can feel immediately after overdose reversal, and the challenges this presents for service engagement:

Overdoses are not necessarily a teachable moment…I’m slipping off into a nice fucking warm nod, I’m high, and I wake up and I’m fucking sick… and I’m trying not to shit my pants, and not throw up, and I have someone with a badge or EMS leaning over me [saying], “Shit, what’s your name?” You know, it’s a traumatic event.

Several professional participants also highlighted that the way responders treat survivors immediately post-overdose can significantly influence survivors’ decisions about whether to engage in discussions about treatment and service options, commenting specifically on how disrespect, stigma, and judgment from overdose responders and medical personnel deter engagement. As a harm reduction service provider explained:

Not everybody responding to these to overdoses is going to treat our population how they deserve to be treated, with respect and dignity, like a human being. So a lot of people don’t want to [be transported] to [EDs], they don’t want to go in the ambulance, because they feel like they’re going to be talked down to.

An SUD treatment program manager similarly described how survivors could be deterred from engaging with service providers: *“If you’re treated for overdose [and] you feel that you were significantly judged, that’s going to make [you] want to run away from the whole scene; you’re not going to want to deal with that.”*

##### Limitations of standard processes and related recommendations.

In the study setting, a standard overdose response involves calling first responders who typically administer naloxone to reverse the overdose and then offer transport to a local ED for additional medical monitoring and stabilization. Several professional participants described aspects of standard overdose response processes that reduce responders’ ability to effectively discuss or connect survivors to SUD treatment or other services. First, professionals highlighted that overdose response in Boston is often handled by police, firefighters, or EMS personnel who may be associated with mistrusted institutions responsible for violence and mistreatment towards marginalized communities. As a residential SUD treatment program staff member said, *“People don’t trust the police, right? They don’t want anything to do with them.”* A SUD service navigation program manager explained that this mistrust is especially prominent in communities of color:

Depending on who responds, [there may be an] ambulance…police may have arrived, and you know, police officers—or just uniforms in general—are not friendly for people of color, for the Latin community. So that may trigger something so people may not be as willing to go and seek treatment after they overdose.

Because of this mistrust, a drug user organization leader recommended having a person trained in harm reduction approaches present during overdose response encounters in addition to or instead of police:

The way places are doing it right now [is] bad, like involving police, not good…When somebody is revived, it’s critical to have harm reduction services in place, because when they come to, two things usually happen: they’re in withdrawal, they’re angry…and a lot of times they’re scared… It’s crucial to have a harm reductionist there…[even] a five-minute exchange, trust starts to build, and now this person feels seen, they feel heard.

Second, professionals explained how EMS and EDs are structured to manage acute medical concerns rather than foster relationship-building or have in-depth discussions about longer-term treatment plans. As one EMS provider said, *“Sometimes the only option you have is an emergency room. I’d posit that’s not the best place for someone who is looking for services to go. Emergency rooms are geared to [short] visits, not long-term care.”* The SUD treatment program manager quoted above suggested having more non-EMS overdose responders, such as people trained in social work or harm reduction, whose primary aim could be providing support and referrals to services. This participant felt that this would be more effective than expecting EMS to spend additional time doing so, because within the current system structure, *“If [an] ambulance pulls up, they’re coming to do a job, [and] their job is specific.”* Another EMS provider made a similar recommendation when describing a unique EMS community engagement unit, comparing it to more traditional field units that typically respond to emergencies:

The [community engagement] unit can sit and have a 30- or 35-minute conversation with someone to try to convince them to [access] services, [but] the field units don’t have that availability, and we don’t have enough field units. And the dispatchers [are] trying to get 911 calls off their booth. What I would like to see, in a perfect world, [is] our [community engagement unit]; I’d like to see [more of them].

## Discussion

To inform efforts to engage overdose survivors in evidence-based treatment and other services to prevent subsequent fatal overdose, we sought to understand the immediate post-overdose experiences of survivors and service providers in Boston. Overdose survivors, who were primarily recruited from an area with a high concentration of overdose and public drug use, described physical and emotional distress after overdose reversal that impeded their desire and ability to engage with service providers; regardless, some participants wished to be offered support and expressed disappointment when this did not happen. Though some survivors had positive interactions with overdose responders, others experienced disrespect and stigma, as noted in prior research (Hawk et al., 2021; Neale et al., 2020). Professionals confirmed survivors’ perspectives with stories from their own professional experiences, highlighting how trauma and stigma can reduce survivors’ willingness to accept service information and referrals. They also emphasized the importance of systemic challenges and provided recommendations for enhancing harm reduction involvement and community engagement in overdose response. It is important to note that we did not observe any significant differences in themes across the different racial and ethnic groups that were included in the overdose survivor study sample. Additional research is needed to understand if there are unique experiences for specific racial or ethnic groups that did not emerge in our study.

Models of overdose response vary considerably by setting, region, and across countries. Responding to an overdose in a rural private residence with limited local addiction infrastructure is distinct from a response within an overdose prevention site. The strength of evidence for specific models also varies from robust (e.g., overdose prevention sites) to emerging (e.g., post-overdose outreach). Nonetheless, these findings can help inform strategies to effectively engage overdose survivors across settings, including locations where fentanyl has not yet become the dominant opioid in the non-prescribed drug supply. First, overdose first responders and related protocols should foster an environment in which survivors can be physically and mentally able to participate in conversations with service providers. This aligns with advocacy with and by people who use drugs around the world calling for compassionate care and overdose response standards utilizing specific techniques to minimize opioid withdrawal and the corresponding negative side effects, and to create a calm and supportive environment that is conducive to providing further care (Greer et al., 2016; Russell et al., 2024). Monitoring to ensure adherence to and efficacy of this standard is critical. Additionally, emergency providers, staff who respond to overdoses, and people who work with survivors should be trained in trauma-informed approaches and resourced appropriately to spend time supporting survivors with emotional and mental distress in addition to physical stabilization needs (“Liberating Methadone: A Roadmap for Change - Conference Proceedings and Recommendations,” 2024). As suggested by a professional participant, in settings with many overdoses, harm reduction outreach specialists could partner with emergency responders for this purpose. Equipping emergency responders with leave behind naloxone to engage individuals and their social networks is another more feasible approach (Scharf et al., 2021). Organizations whose staff respond to overdoses should also implement strategies to reduce stigma towards people who use drugs, such as interventions that include interactions with people in recovery (Bielenberg et al., 2021; Murray et al., 2024). In addition to training and interventions to reduce stigma and leave behind naloxone, EMS and other agencies providing overdose response should review protocols with a trauma-informed lens to identify and eliminate practices that could increase trauma, such as clothing being removed without consent (unless medically necessary) or questioning immediately after the patient regains consciousness. Finally, sanctioned overdose prevention centers - available in many European and Canadian cities but only in limited locations in the United States - could address many of the challenges identified in our study, as they are designed to be supportive environments where trained staff can provide compassionate and nonjudgmental overdose response (Harocopos et al., 2022; Samuels et al., 2022; Stevens et al., 2024).

Next, our findings provide support for post-overdose outreach programs that seek to engage survivors in the days following their overdose. For survivors who are unable to participate in conversations with service providers immediately after overdose reversal, these outreach programs can provide an effective touchpoint for follow-up connections to care (Formica et al., 2022; 2021; Kimmel et al., 2023; Xuan et al., 2023). Existing post overdose outreach programs are often comprised of staff from public safety, public health, and harm reduction programs (“PRONTO: Partnerships for Post-Overdose Outreach,” n.d.). Our findings suggest that outreach by public health or harm reduction staff may be more effective than outreach from public safety officials who may be mistrusted, especially in communities of color. Follow-up outreach should also be in addition to, rather than a substitute for, immediate information and referrals. Although these immediate offers of support may not directly result in treatment or harm reduction service uptake, they represent support to survivors, and their absence may diminish survivors’ trust in service providers and their institutions.

Finally, in considering the factors that may affect how responders treat survivors, it is important to recognize that some first responders, emergency providers, and outreach workers, experience emotional fatigue, perceptions of “failure” in achieving SUD service uptake, or burnout from their overdose response efforts (Beaugard et al., 2023; Jozaghi et al., 2018; Nicholson et al., 2024; O’Dare & Atwell, 2023; Patch et al., 2023; Pike et al., 2019; Schoenberger et al., 2024). Consistent with calls from organizations of people who use drugs, both responders (including those who use drugs themselves) and survivors may benefit from overdose response processes that better prioritize person-centered models of care and trust-building as key outcomes (rather than uptake of specific services). This includes understanding each individual’s unique needs, and being able to make connections to social services and other resources (e.g., housing) that survivors may most urgently need (Kimmel et al., 2023), in addition to SUD-specific supports.

### Limitations

This study has limitations. We primarily recruited overdose survivors in a Boston neighborhood with a high concentration of public drug use as well as local SUD services, and recruitment overlapped with the COVID-19 pandemic, which impacted service availability delivery. Additionally, our professional participant sample did not include all agencies or roles involved in overdose response, and our recruitment and eligibility criteria did not focus on peers or other non-professional bystanders who respond to overdoses and likely have unique perspectives (Brandt et al., 2022; Jacka et al., 2024). Further, some themes occurred only among a small number of participants. Thus, findings may not be generalizable to different populations, settings, countries, or time periods. Finally, this analysis was part of a larger study addressing several additional areas of inquiry (Paradise et al., 2024), possibly limiting our ability to comprehensively explore all aspects of the immediate post-overdose experience. In particular, the overdose survivor interview guide was designed to specifically explore experiences and perspectives regarding formalized care systems and professional providers. It was not designed to explore the experience of having an overdose reversed by a peer or community member and how this affects service engagement. Further study may be required to understand this and other topics, such as emotional responses experienced after overdose and their impact on service-seeking, in greater depth or from different perspectives.

### Conclusions

To identify how immediate post-overdose response and follow-up processes can better engage survivors and facilitate connections to services, it is essential to understand the experiences and perspectives of overdose survivors and service providers. This study contributes to the literature by bringing together multiple diverse perspectives on challenges and opportunities in the immediate post-overdose period, including perspectives of overdose survivors who were disengaged from SUD treatment systems as well as professionals who brought a systemic lens. Findings contribute new information about survivors’ emotional responses after overdose and how this may affect engagement in follow-up services, in addition to adding to prior literature regarding physical reactions to overdose reversal and stigma in encounters with service providers. Participants highlighted multiple challenges impeding survivors’ engagement in services immediately post-overdose, and there are corresponding opportunities for improvement. Successful post-overdose outcomes should be defined more broadly than immediate uptake of SUD services, and processes should focus on providing supports and service information in person-centered, trauma-informed, and non-stigmatizing ways that improve survivors’ comfort, reduce feelings of guilt and shame, repair trust in systems, and alleviate provider burnout.

## Figures and Tables

**Table 1:** Sociodemographic and substance use characteristics of opioid overdose survivor participants (*N* = 59).

	N	Percent

**Age group**		
18–29	4	7 %
30–39	26	44 %
40–49	16	27 %
50–59	11	19 %
60 or older	2	3 %
**Gender**		
Cisgender Male	41	70 %
Cisgender Female	15	25 %
Transgender Female	2	3 %
Transgender Male	1	2 %
**Sexual orientation**		
Straight or heterosexual	52	88 %
Gay, lesbian, or homosexual	2	3 %
Bisexual	3	5 %
Something else	2	3 %
**Race/ethnicity**		
Non-Latino/a/x Black	18	31 %
Hispanic or Latino/a/x	23	39 %
Non-Latino/a/x White	18	31 %
**Time living in Boston**		
Less than 6 months	3	5 %
6 months to less than 1 year	2	3 %
1 to 5 years	12	20 %
More than 5 years	28	48 %
Always lived in Boston	14	24 %
**Housing arrangement**		
Home/Apartment	9	15 %
Residential facility (including residential treatment)	5	9 %
Staying with friend or relative	1	2 %
Shelter	15	25 %
Street	28	48 %
Staying between shelter, street, and with relative	1	2 %
**Employment** *		
Volunteering	13	22 %
Employed	3	5 %
Out of work	39	66 %
Unable to work	13	22 %
**Spent time in criminal justice facility**	50	85 %
**Physical health status, past month**		
Excellent	6	10 %
Good	20	34 %
Fair	24	41 %
Poor	9	15 %
**Mental health status, past month**		
Excellent	–	–
Good	15	25 %
Fair	26	44 %
Poor	18	31 %
**Substances used, last month**		
Heroin/fentanyl	55	93 %
Cocaine or crack	47	80 %
Sedatives	39	66 %
Cannabis	38	64 %
Amphetamine or prescription stimulants, not prescribed	25	42 %
Alcohol	21	36 %
Methadone, not prescribed	13	22 %
Other prescription opiates, not prescribed	16	28 %
**Overdoses, past three months**		
Median (Interquartile Range)	2.0 (2.5)	–
1–2	41	70 %
3–5	13	22 %
6–9	1	2 %
10 or more	4	7 %
**Overdoses, past year**		
Median (Interquartile Range)	4.0 (7.0)	
1–2	18	30 %
3–5	17	29 %
6–9	11	19 %
10 or more	13	22 %

* Participants could select more than one employment category.

**Table 2:** Race/ethnicity, organization, and role of professional participants (*N* = 28).

	N	Percent

**Race/ethnicity**		
Non-Latino/a/x Black	8	29 %
Hispanic or Latino/a/x	7	25 %
Non-Latino/a/x White	12	43 %
Unknown	2	7 %
**Organization type** *		
City of Boston	6	21 %
Community Health Center	4	14 %
Drug User Organization	2	7 %
Emergency Medical Services or Fire Department	3	11 %
Other Emergency Services Program	1	4 %
Harm Reduction Program	4	14 %
Homeless Shelter	1	4 %
Hospital	2	7 %
SUD Treatment Program (Outpatient)	7	25 %
SUD Treatment Program (Acute Treatment Services or Residential Treatment	5	18 %
None (Community Leader)	1	4 %
**Role** *		
Advocate/Activist	3	11 %
First responder	3	11 %
Leader/Policy maker	2	7 %
Licensed service provider (front line staff)	2	7 %
Licensed service provider (manager)	1	4 %
Other service provider (front line staff)	6	21 %
Other service provider (manager)	10	36 %
Physician (MD)	4	14%

* Participants could represent more than one organization and/or have more than one role.
